# Engineering Light-Control in Biology

**DOI:** 10.3389/fbioe.2022.901300

**Published:** 2022-04-28

**Authors:** Armin Baumschlager

**Affiliations:** Department of Biosystems Science and Engineering (D-BSSE), ETH Zürich, Basel, Switzerland

**Keywords:** optogenetics, light-control, photosensors, synthetic biology, bioengineering

## Abstract

Unraveling the transformative power of optogenetics in biology requires sophisticated engineering for the creation and optimization of light-regulatable proteins. In addition, diverse strategies have been used for the tuning of these light-sensitive regulators. This review highlights different protein engineering and synthetic biology approaches, which might aid in the development and optimization of novel optogenetic proteins (Opto-proteins). Focusing on non-neuronal optogenetics, chromophore availability, general strategies for creating light-controllable functions, modification of the photosensitive domains and their fusion to effector domains, as well as tuning concepts for Opto-proteins are discussed. Thus, this review shall not serve as an encyclopedic summary of light-sensitive regulators but aims at discussing important aspects for the engineering of light-controllable proteins through selected examples.

## 1 Introduction

The use of light for deciphering and controlling biological processes has become an enabling methodology for basic research and biotechnological approaches alike. Although the definition of the term “optogenetics” itself is under discussion (Adamantidis et al., 2015), it was first mentioned in the context of light imaging and manipulation of neuronal circuits (Deisseroth et al., 2006). In a broad definition, optogenetics can be seen as the use of light-sensitive genetically encoded elements, which fulfill diverse functions. Key aspects that make the use of light attractive for biological research and bioproduction are the ability for temporal as well as spatial application of light (Baumschlager and Khammash 2021). In addition, light inputs might be less invasive and more orthogonal in non-photosensitive cells compared to the addition of chemical inducers. Furthermore, the replacement of small-molecule inducers with light could reduce costs in large-scale bioprocesses (Baumschlager and Khammash 2021).

In this review, the focus is set on engineering approaches of biological components for inter- and intramolecular light regulation of diverse cellular functions. This builds on and extends our recent review (Baumschlager and Khammash 2021), in which we describe the types and design principles of photoactivatable proteins. Here, I delve further into practical engineering aspects, that go beyond general design aspects. First, important considerations for the availability of chromophores in biological systems are discussed. Then, studies are presented that use either native light-sensing regulators or employ different strategies for the creation of novel optogenetic hybrid proteins and describe their underlying regulation principles. This will serve as the basis to investigate the effects of photosensory domain modifications. The review is concluded by discussing examples that optimize Opto-protein functions, especially through their linking regions and via tuning of their intracellular concentration. All of these aspects might be helpful for the construction, implementation, and optimization of optogenetic regulation in cells.

Although intended to be kept at a minimum, some terminology specific to the field of optogenetics will be used in this review. These include “dark state” or “leakiness”, which refer to the residual activity of the Opto-protein in the uninduced state, typically when no light input is applied. The difference between this dark state and the light-activated state of the Opto-protein constitutes the “fold-change”. “Photosensor”, “photoreceptor” or “photoregulator” refers to proteins or protein domains, in which light absorption, typically from a specific wavelength range, causes a structural change in the protein used for different cellular regulation strategies.

## 2 Availability of Chromophores

Chromophores are light-sensing molecules or chemical moieties of photosensitive protein domains, that define the absorption range of electromagnetic wavelengths (Baumschlager and Khammash 2021). Light absorption of the chromophore causes changes in the protein structure of photosensitive domains through different means, such as an oxidation state change, structural changes of the chromophore, and/or its interactions with the apoprotein (Spiltoir and Tucker 2019; Baumschlager and Khammash 2021). Therefore, not just transfer of the photosensitive protein domain itself, but also the availability of the chromophore in the cell must be considered when choosing the photosensitive domain for an Opto-protein design.

**FIGURE 1:**
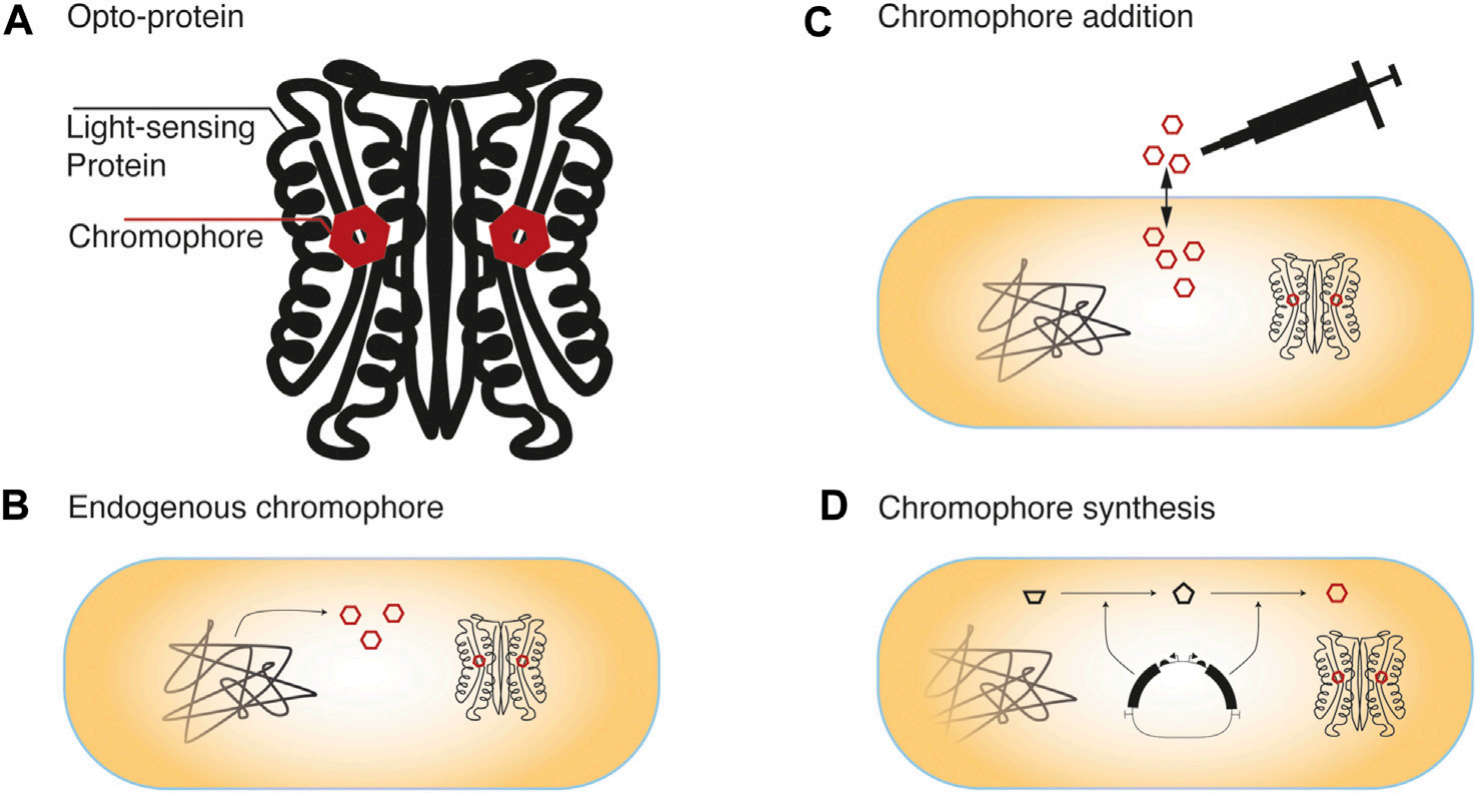
Chromophore availability in cells **(A)** Light sensing proteins contain either light-absorbing tryptophane conformations or bound small-molecule chromophores **(B)** Depending on the host organism and the photosensitive protein, the chromophore might be available in the cell through its native metabolism. Otherwise, availability of the chromophore can be realized through **(C)** external supplementation of the chromophore or its precursors, which requires their diffusion or active uptake into the cell, or by **(D)** transferring the genes necessary for its synthesis into the cells of interest.

Some of the most commonly used photosensitive proteins employ either photosensitive tryptophan conformations within their domains or a bound flavin-, cobalamin- or tetrapyrrole-based chromophore (Figure 1). Both tryptophan, being a proteinogenic amino acid, and flavin-chromophores (flavin mononucleotide FMN, flavin adenine dinucleotide FAD), being important coenzymes in redox reactions, are essential components in most cells (Figure 1B). However, cobalamin- or tetrapyrrole-based chromophores are not ubiquitously available, and therefore have to be supplied depending on the organism of interest. This supplementation can either be realized through exogenous addition (Figure 1C) or by genetically engineering synthesis capabilities into the host strain (Figure 1D). Other examples apart from the mentioned and commonly used chromophores that will be discussed in more detail hereafter, include the photoactive yellow protein (PYP) that incorporates *p*-coumaric acid, which is not present in most cells. However, it can be added exogenously or its biosynthesis can be genetically engineered by introducing a tyrosine ammonia lyase and *p*-hydroxycinnamic acid ligase into the cell (Kynd et al., 2003). Also for possible other chromophores that are not discussed in the following section, it has to be considered if the chromophore is already available in the cell, needs to be supplied exogenously, or can be produced through the incorporation of a metabolic pathway. (Figures 1B–D).

### 2.1 Proteinogenic Amino Acid Tryptophan

An example of photoreceptors in which the protein itself acts as light-sensing moiety is UVR8 from *Arabidopsis thaliana*. The protein forms a homodimer in its dark state and dissociates upon UV-B radiation (Rizzini et al., 2011). The released UVR8 monomers can then bind to its interacting protein COP1 (Rizzini et al., 2011). Absorption of UV-B light is mediated through a cluster of tryptophans (W233, W285, and W337), in the center of the protein, which causes a rearrangement and the release of intermolecular hydrogen bonds between the UVR8 homodimers (Wu et al., 2012). Tryptophan shows an absorption maximum at around 280 nm in solution (Rizzini et al., 2011). It is the least abundant of the canonical amino acids and is more conserved than other amino acids in proteins (Alkhalaf and Ryan 2015). Thus, being a proteinogenic aromatic amino acid, additional supply or synthesis of a separate small molecule chromophore is usually not required.

### 2.2 Flavin-Chromophores

Photoreceptors that use cellular flavins (FMN, FAD) as chromophores belong to Cryptochromes (Brautigam et al., 2004), BLUF (blue-light sensors using FAD) (Gomelsky and Klug 2002), and LOV (Light Oxygen Voltage) domains (Christie et al., 2012). Specific examples are cryptochromes CRY1 and CRY2 (Brautigam et al., 2004), and BLUF domain PixD (Yuan and Bauer 2008), which bind FAD. In LOV domains, both FAD- and FMN-binding domains were identified. For example, FMN functions as a chromophore in *Avena sativa* phototropin 1 LOV2 (Alexandre et al., 2009), and FAD is bound in *Neurospora crassa* photoreceptor Vivid (VVD) (Zoltowski et al., 2007). In *E. coli*, FMN synthesis is regulated on the transcriptional level by the FMN riboswitch (Abbas and Sibirny 2011). Interested readers are referred to a review by Abbas and Sibirny for an overview of flavin synthesis regulation in different organisms (Abbas and Sibirny 2011). Interestingly, Hühner et al. found that riboflavin, FAD, and FMN concentrations vary between mammalian cell lines, where riboflavin (3.1–14 amol/cell) and FAD (2.2–17.0 amol/cell) are the predominant flavin species (FMN: 0.46–3.4 amol/cell) (Hühner et al., 2015). They also concluded that native flavin contents should be sufficient for synthetic biological applications, but could be limiting for very strong overexpression, which might have to be considered in Opto-protein designs (Hühner et al., 2015).

### 2.3 Cobalamin-Based Chromophores

Cobalamin-binding domains (CBDs) are photoreceptors that respond to green light. An example is the CarH photoreceptor dimer that binds coenzyme B12 or 5′deoxyadenosylcobalamin (AdoCbl) as chromophore (Takano et al., 2015). Only certain bacteria and archaea are capable of vitamin B12 synthesis. For example, the biotechnologically highly relevant bacterium *E. coli* is incapable of B12 synthesis but can transform it to AdoCbl when supplied in the medium (Ortiz-Guerrero et al., 2011). Also mammalian cells can take up cobalamin and transform it to AdoCbl (Quadros and Sequeira 2013). As an example of typical chromophore concentrations in mammalian cell culture, 20 µM AdoB12 was used in experiments with HEK-293 cells (Schneider et al., 2021). The authors noted, that AdoB12 is not stable in DMEM complete medium and will slowly degrade with a half-life time of approximately 24 h and subsequently suggested higher initial concentrations of AdoB12 for longer-lasting experiments. Depending on the organism used and the specific application it might be required to test appropriate chromophore concentrations. For example, different concentrations were used for light-controlled CarH-mediated cell-cell adhesion of MDA-MB-231 cells. Nzigou Mombo et al. exploited that surface-displayed CarH requires AdoB12 to form a tetramer, and consequently used the AdoB12 concentration in the media (0.5–10 μm) as a tuning knob for the ratio of active CarH. Cells started clustering at concentrations of 1 µm AdoB12 and the area of the clusters increased with the concentration (Nzigou Mombo et al., 2021). Apart from exogenous supplementation, it was shown that *de novo* engineering of vitamin B12 synthesis in *E. coli* via an aerobic biosynthetic pathway is possible (Fang et al., 2018). Therefore, cobalamin supplementation, as well as uptake and transformation capabilities of the host organism, have to be considered in the design of CBD-containing Opto-proteins.

### 2.4 Tetrapyrrole-Based Chromophores

Phytochromes (Phy) are photosensitive proteins that usually incorporate a tetrapyrrole chromophore and absorb a wide range of wavelengths. Phytochromes utilize different tetrapyrrole-based chromophores depending on the origin of the photoreceptor. For example, phycocyanobilin (PCB) and phycoerythrobilin (PEB) are found in light-sensing proteins in algae, PCB and biliverdin (BV) are chromophores employed in bacteria and phytochromobilin (PΦB) is found in plants (Mukougawa et al., 2006; Baumschlager and Khammash 2021). Interestingly, some photoreceptors such as *Arabidopsis thaliana* phytochrome B (PhyB) can bind both PΦB and PCB. Which of these chromophores are bound influences the absorption maxima of both the red-absorbing (Pr) as well as the far-red-absorbing (Pfr) state (Burgie et al., 2017). It was further discovered, that PCB stabilizes the Pfr state of PhyB compared to the native PΦB (Burgie et al., 2017).

Exogenous supplementation of PCB is possible, as it is taken up by yeast or mammalian cells (Shimizu-Sato et al., 2002). However, the effort of its chemical synthesis and its low stability have to be taken into consideration (Müller et al., 2013). Purification from *Spirulina* algae can be an economical option for the extraction of PCB (Scheer 1984; Levskaya et al., 2009; Toettcher et al., 2011). Alternatively, commercially available PCB might be applicable. However, contaminants of commercial preparations were described to lead to constitutive activity at high levels of the photoregulator and show considerable autofluorescence at red and near-infrared wavelengths. Such impurities can be separated from active PCB through high-performance liquid chromatography (HPLC) (Goglia et al., 2017). Purified PCB was used at a concentration of 10 µM in mammalian cells (Goglia et al., 2017). Similarly, 10 µM (Milias-Argeitis et al., 2011) and 25 µM (Shimizu-Sato et al., 2002) PCB was used as chromophore concentrations for experiments involving PhyB/PIF in *Saccharomyces cerevisiae* as two examples. Also here, the chromophore concentration should be adapted to the organism and experimental conditions. Due to chemical synthesis/purification and low chromophore stability, intracellular production of PCB is usually preferred for long-term experiments. For this, PCB synthesis in *E. coli* was genetically engineered using two enzymes (heme oxygenase (H O 1), phycocyanobilin:ferredoxin oxidoreductase (pcyA)) from cyanobacterium *Synechocystis sp.* PCC6803 for conversion of heme to biliverdin IX (BV) and BV to PCB (Gambetta and Lagarias 2001). Also PΦB could be synthesized using Ho1 and a truncated PΦB synthase enzyme (Arabidopsis HY2) (Mukougawa et al., 2006). Similarly, PEP was synthesized from BV using dihydrobiliverdin:ferredoxin oxidoreductase (PebA) and PEB:ferredoxin oxidoreductase (PebB) (Mukougawa et al., 2006). In mammalian cells, the two enzymes Ho1 and PcyA were sufficient for PCB production (Müller et al., 2013), which was further improved through ferredoxin (Fd) and Fd-NADP + reductase (Fnr) coexpression (Uda et al., 2017). The concentration of available BV, such as used in NIR-responsive bacterial phytochrome BphP1, depends on the particular tissue of mammalian cells (Redchuk et al., 2017, 2020). Thus, cofactor supplementation and/or its synthetic intracellular production might have to be considered when using phytochrome domains.

## 3 Native Light-Sensing Regulators and Novel Chimeric Proteins

**FIGURE 2:**
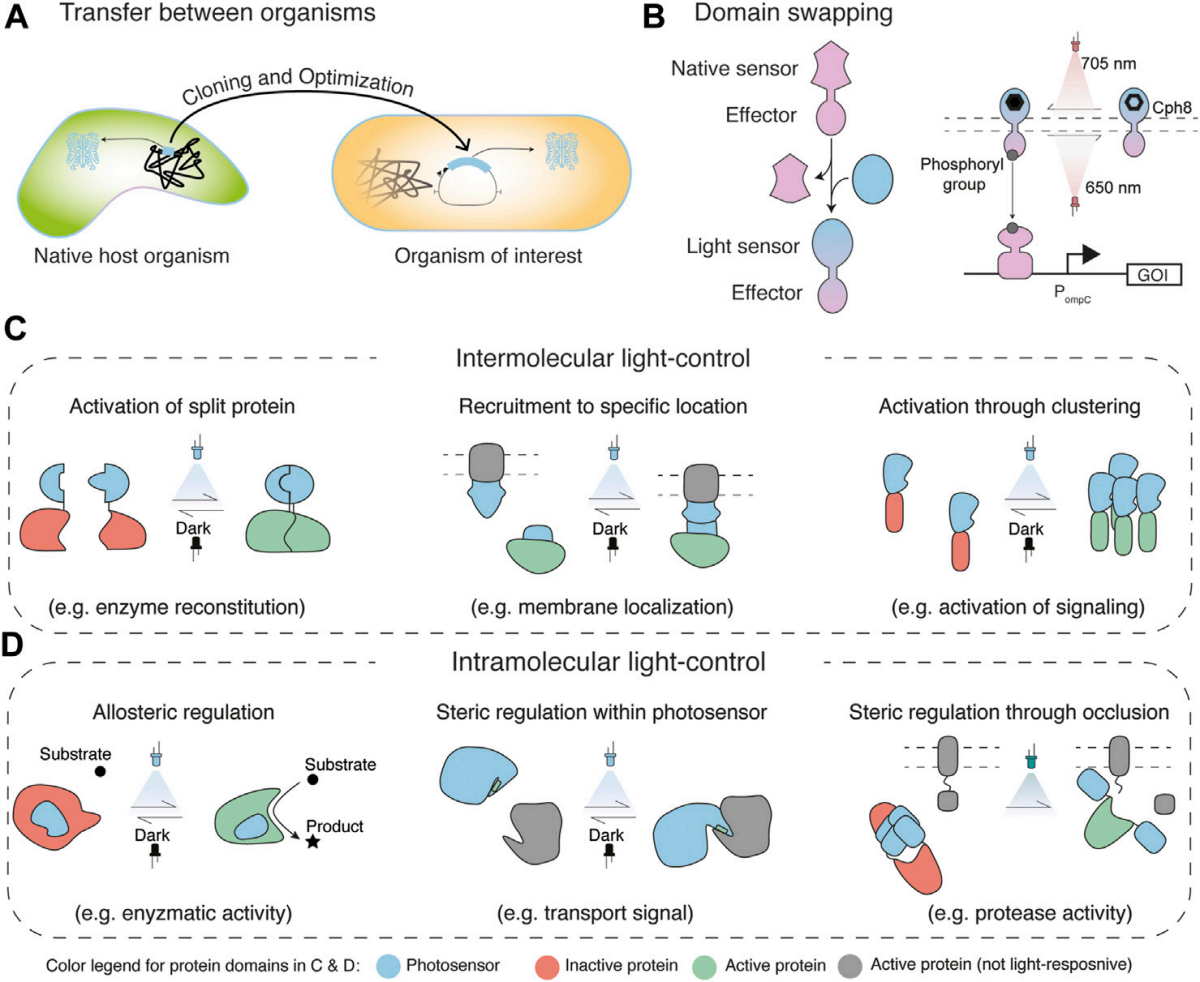
Engineering strategies for light regulation **(A)** Transfer of light-sensing regulators and circuits from native host organisms into an organism of interest requires compatibility of the respective function and optimization for the new host **(B)** Change of the native sensing function of a protein to sensing of light through domain swapping (left), which was shown for the transformation of an osmoregulation bacterial two-component system (TCS) to a synthetic light-regulatable TCS (Levskaya et al., 2005). An intuitive way to categorize strategies for the creation of novel Opto-proteins is into intermolecular **(C)** and intramolecular light-control **(D)** (Baumschlager and Khammash 2021). Intermolecular light-control functions through proximity and distance of proteins. Thus, it usually comprises two or more interacting proteins, whose binding/distance can be regulated with light. Such regulation can be achieved through the reconstitution of inactive protein domains (e.g. split fragments in Opto-T7RNAP (Baumschlager et al., 2017); **(C)**, left), the recruitment of an active protein to a location where it exerts function (e.g. membrane localization for control of phosphoinositide 3-kinase activity (Toettcher et al., 2011); **(C)**, middle), or activation of a cellular function through clustering (e.g. activation of cell signaling with optoWnt (Rosenbloom et al., 2020); **(C)**, right). In contrast, intramolecular control involves the development of a single protein chimera that comprises a light-responsive and an effector function. Regulation can be achieved allosterically in which light absorption leads to a structural change in the protein that changes the activity of the effector domain (e.g. enzymatic activity in yeast isocitrate dehydrogenase (Chen et al., 2021); **(D)**, left), steric blocking/unblocking of the function of the effector (e.g. exposure of a transport signal in the light-inducible nuclear export system (LEXY) (Niopek et al., 2016); **(D)**, middle), or steric regulation through occlusion (e.g. active site blocking of a protease (Zhou et al., 2012); **(D)**, right).

This section discusses strategies for the implementation of light regulation in cells. These approaches either make use of native light regulators (Figure 2A) or create synthetic chimeric light-sensing proteins (Figure 2B–D). The transfer of light-sensing regulators from their natural host to an organism of interest might be the most straightforward approach to implement light-control of a specific function (Figure 2A). Such a transfer may involve optimization of the codon usage and adaptation of the expression system (promoters, translation initiation, etc.) for the new host along with the targeting of the light regulator towards the function of interest.

### 3.1 Transfer of Photoregulators to an Organism of Interest

An example for successful transfer of light-sensitive systems from one organism to another was demonstrated for the two-component gene expression system (TCS) CcaS/R from *Synechocystis sp.* PCC 6803 (Hirose et al., 2008; Tabor et al., 2011). Like most TCSs, CcaS/R comprise a sensor kinase (CcaS) and a response regulator (CcaR) (Schmidl et al., 2019). CcaS binds the chromophore PCB and shows green light-induced activation (535 nm) of autophosphorylation. This activation can be reversed with red-light (672 nm) or through thermal reversion. Autophosphorylation of CcaS enables phosphotransfer to the cognate response regulator CcaR, which then induces the expression of cpcG2. Tabor et al. successfully transferred the CcaS/R transcription regulation system from *Synechocystis* into *E. coli* (Tabor et al., 2011). In brief, both sensor kinase and response regulator were expressed along with the enzymes for intracellular PCB production. This allows for green-light induced expression of the desired gene of interest from the cpcG2 promoter. Important optimization steps included promoter engineering and expression level tuning, which will be discussed in more detail in a subsequent section.

Also light-induced single component systems were effectively moved from a natural host organism to an organism of interest. An example for this is the light-activated transcription factor EL222 from the marine bacterium *Erythrobacter litoralis* HTCC2594 (Rivera-Cancel et al., 2012; Zoltowski et al., 2013). Blue light drives the reorientation of sensory N-terminal LOV and the C-terminal NatL/LuxR-type helix−turn−helix (HTH) effector domains of EL222, which then bind to a cognate EL222 DNA binding region to allow for photoactivation of gene transcription through light-dependent DNA-binding (Rivera-Cancel et al., 2012; Zoltowski et al., 2013). Inactive EL222 in the dark is monomeric, which is stabilized by inhibitory contacts of the LOV with the HTH domain. The light-induced conformational change in the LOV domain releases these contacts and frees the HTH domain, which allows for dimerization of two EL222 proteins and subsequent binding to the 12 bp EL222 binding sequence (Rivera-Cancel et al., 2012; Zoltowski et al., 2013). Removal of the light-stimulus leads to reversion to the monomeric form and release of DNA-binding. This light-induced DNA-binding and transcriptional activation mechanism was successfully transferred from the native host *E. litoralis* to *E. coli* to control transcriptional activation as well as repression (Jayaraman et al., 2016). For this, EL222 was expressed in *E. coli* and corresponding EL222-responsive promoters were engineered. In the promoter (P_BLind_), the EL222 binding region replaced the lux box of a luxI promoter to create light-induced activation. In another promoter (P_BLrep_), the binding region was placed between consensus −35 and −10 regions of RNAP for light-induced repression, so that EL222-binding presumably impeded the binding of RNAP (Jayaraman et al., 2016).

In both discussed cases, no modifications of the light-sensitive proteins themselves (CcaS/R or EL222) were necessary and their native function could be preserved and redirected for its use in a new host organism.

### 3.2 Intermolecular Spatial Regulation

If certain functionalities or properties cannot be achieved through the transfer of native light-sensing proteins, then synthetic chimeric light-sensitive proteins have to be engineered. Strategies for such engineering approaches usually rely on the creation of hybrid proteins comprising effector domains that contain cellular functionalities and photo-responsive sensing domains for light regulation.

A widely used design strategy for Opto-proteins is light-mediated spatial regulation. Typically, the absorption of light by the chromophore/photosensitive domain leads to an allosteric change within the protein domain. This exposes an interface that can interact with other photosensory or interaction domains (Figure 2C). Early examples for such regulators were based on two-hybrid systems (Ni et al., 1999; Shimizu-Sato et al., 2002), which have been extensively used to discover and investigate protein-protein interactions (Young 1998). Here, the Gal4 transcription factor is split into two entities, a DNA-binding domain, and an activation domain. For Opto-protein designs, each of the two split parts was then genetically fused to either PhyB or phytochrome interaction factor 3 (PIF3). The binding of PhyB and PIF3 in turn can be controlled with light (Ni et al., 1999; Shimizu-Sato et al., 2002). Light-controlled reassembly of the transcription factor, thus spacial recruitment of the activation domain to the Gal4 promoter, activates transcription of a gene of interest via polymerase recruitment. Since this initial work was published, numerous other split proteins were engineered which involve the use of different photoreceptors also using Gal4 (Hughes 2018) or other proteins (e.g. Cas9 (Nihongaki et al., 2015; Polstein and Gersbach 2015), Cre-recombinase (Kennedy et al., 2010; Sheets et al., 2020), polymerases (Figure 2C, left) (Baumschlager et al., 2017) and others (Hughes 2018)).

Intermolecular spatial regulation was further employed for the recruitment of effector domains to a location where they exert a specific function (Figure 2C, middle). As such, Levskaya et al. engineered light-control for the activation of Rho-family G-protein signaling proteins through their recruitment to the plasma membrane (Levskaya et al., 2009). This work is discussed in more detail in section 4.3.

Also protein oligomerization of multiple individual Opto-proteins into functional multimers (Figure 2C, right) was demonstrated through light-controlled spatial assembly or dissociation of photosensitive domains which are fused to effector proteins (Bugaj et al., 2013; Taslimi et al., 2014). Cry2 is an oligomerizing photosensitive protein (Mas et al., 2000) that was used for optogenetic protein clustering. In general, oligomerization is a mechanism for cellular regulation, which includes cell signaling and enzymatic activities (Mammen et al., 1998). Bugaj et al. demonstrated optogenetic clustering of the LRP6 C-terminal domain (LRP6c) fused to Cry2, to activate β-catenin signaling as one such example. Oligomerization was shown to be necessary for activation of LRP6s as dimerization did not induce a β-catenin transcriptional response (Bugaj et al., 2013). Taslimi et al. used a clustering-optimized Cry2 variant (CRY2olig) to reversibly detect protein-protein interactions using Light-Induced Co-clustering (LINC). The authors also used inducible-clustering of CRY2 to perturb protein interactions, by showing the conditional light-dependent disruption of endocytosis with a CRY2-clathrin light chain (CLC) fusion, or manipulation of actin polymerization with a CRY2 fusion to the Nck SH3 domains or the VCA domain of N-WASP (Taslimi et al., 2014). Inhibition through clustering was also demonstrated by Lee et al. through “light-activated reversible inhibition by assembled trap” (LARIAT), which, in contrast to the previously described work, used wild-type CRY2 for inducible cluster formation with multimeric proteins (MP) containing a fusion of CIB1 to Ca2+/calmodulin-dependent protein kinase IIα (CaMKIIα), which self-assembles into an oligomer with 12 identical subunits (Lee et al., 2014). Here, light-induction causes CRY2 oligomerization and binding to CIB1, which can be used to inactivate CRY2-fused effector proteins. As one example, Lee et al. fused CRY2 to signaling protein Vav2, a guanine nucleotide exchange factor that activates Rho small GTPases and induces membrane protrusion by translocation to the plasma membrane. Light-induced trapping of CRY2-Vav2 in clusters led to retraction of lamellipodia, thus allowing local control of membrane protrusion and retraction with light.

### 3.3 Intramolecular Allosteric Regulation

In the previous examples, allosteric changes of photosensory domains were used for regulating interactions between different protein domains. However, light-mediated allosteric changes can also directly change the function of the protein that the domain is inserted in. In a general definition, allosteric regulation typically involves protein rearrangements that occur at a site that has some distance to the active site which is transmitted within the protein so that it increases or decreases its activity (Laskowski et al., 2009). This differs for example from competitive inhibition where an inhibitor binds directly to the active site and prevents access to it (Laskowski et al., 2009). Allosteric changes in the regulated protein might lead to either activation via opening the access to an active site, or rearrangements within the protein to form a functional active site (Figure 2D, left). Similarly, allostery can also lead to inversed effects, meaning an inactivation of an enzyme via active site closing or active site distortion (Laskowski et al., 2009). Some light-sensitive proteins can undergo relatively large conformational changes upon light stimulation, which were successfully used to directly activate or inactivate the function of different proteins.

An example for allosteric intramolecular light-control of an enzyme was shown by Chen et al. (Figure 2D, left). In this work, the photosensitive LOV2 domain was inserted into the two-subunit NAD^+^-specific *S. cerevisiae* IDH to enable light control of the metabolic flux through the citric acid cycle in budding yeast (Chen et al., 2021). Using a computational approach, insertion of a LOV2 domain was predicted so that either the dark or the light-induced structure selectively preserves the native and catalytically active conformation of IDH (Chen et al., 2021). This strategy was built upon pioneering work from Dagliyan et al. in which an approach for the use of light-sensitive domains, for allosteric conversion between a natural active conformation and an inactive state, was described (Dagliyan et al., 2016).

Another approach for the use of light-controlled allostery incorporates cellular recognition signals into a functional structure of photoresponsive domains (Figure 2D, middle). An early example of this is the light-inducible nuclear export system (LEXY), which comprises a modified LOV2 domain that has a nuclear export signal incorporated into its C-terminal Ja helix (Di Ventura and Kuhlman 2016; Niopek et al., 2016). This exploits that the Ja helix from *As*LOV2 displaces from the PAS core after the light-stimulation (Harper et al., 2004; Eitoku et al., 2005; Baumschlager and Khammash 2021). This displacement was used for controlled exposure and thus unmasking of the transport signal. The exposed signal can then be recognized by cellular components, such as the nuclear CRM1 (exportin-1) receptors (Wehler et al., 2016). In this case, the signal was incorporated into the photosensitive protein, which might only be applicable for shorter recognition signals. However, also in larger proteins, steric blocking of interaction or active sites was shown as an alternate strategy for allosteric light-regulation (Wu et al., 2009).

Further, light-dependent dissociation and association of photoresponsive domains was also applied for intramolecular regulation. For this, Zhou et al. used a mutant of the photochromic fluorescent protein Dronpa (Dronpa145N), in which cyan illumination induces a shift from cyan-absorbing to violet-absorbing species and a loss of green fluorescence. This is accompanied by a shift from tetrameric toward monomeric Dronpa proteins. Fusion of two Dronpa145N domains to the N- and C-terminus of the hepatitis C virus (HCV) NS3-4A protease rendered the activity of the protein inactive in the dark (Zhou et al., 2012). To visualize protease activity, mCherry was tethered to the membrane via a CAAX-box and contained the cleavage site of HCV polypeptide between the membrane anchor and the fluorescent protein. Off-switching of the Dronpa fluorescence led to uncaging of the enzyme, presumably due to Dronpa monomerization, which was observed by the release of mCherry from the plasma membrane (Figure 2D, right). The authors mention that this caged protein design does not require precise linkages and therefore should be easily generalizable.

### 3.4 Exchange of Sensory Domains

While the approaches discussed above rely on adding a sensory function to an effector domain, implementation of light-control into a protein was also shown using domain swapping strategies. Here, a specific domain (e.g. small molecule sensing domain) of a protein of interest is swapped with a light-sensing domain (Figure 2B) The regulation mechanism of the original protein (e.g. protein dimerization, recruitment, etc.) should be compatible with the mechanism of the light-sensing domain in order to create functional chimeras. This allows one to retain downstream elements of the native sensing molecule, leaving for example the signaling to a response regulator, thus its function, unchanged, while transforming the sensing property to light.

An early example for such a domain swapping strategy was shown with the engineering of a light-sensitive two-component system in *E. coli* (Levskaya et al., 2005). The *E. coli* TCS EnvZ–OmpR regulates porin expression in response to an osmotic shock (Utsumi et al., 1989), which was used as the basis for the chimera engineering. Generally, TCSs are the largest family of multi-step signal transduction pathways. They show a high degree of exchangeability between their sensor kinase and response regulator domains and are thus interesting targets for domain swapping. The sensor kinases typically contain a variable N-terminal sensor domain linked to a C-terminal histidine-kinase domain (Schmidl et al., 2019). To transform the EnvZ–OmpR TCS into a light-sensitive transcription regulator, first the photosensory domain of *Synechocystis sp.* PCC 6803 Cph1, a red/far-red sensing photoreceptor from the phytochrome family, was structurally aligned with EnvZ, the sensor of the TCS, to identify potential crossover points. Cph1 was then fused to the EnvZ histidine kinase domain at the crossover points which created a functional chimera. In this newly created protein, red light inhibits autophosphorylation of the sensor protein, which turns off gene expression from the ompC promoter by OmpR. Similarly, Ohlendorf et al. created the blue-light-repressed histidine kinase YF1 by swapping the two PAS domains of the sensor kinase *Bradyrhizobium japonicum* FixL for the light-sensing LOV domain of *Bacillus subtilis* YtvA (Ohlendorf et al., 2012). This blue-light controllable TCS steers gene expression from the fixK2 promoter through light-controlled changes of the phosphorylation state of the cognate response regulator FixJ (Ohlendorf et al., 2012).

Also within engineered Opto-proteins, swapping of photosensitive domains can be achieved. To increase the chances of success, the light regulation mechanisms and the structural properties of the photosensory domains should be similar. For example, different LOV domains could be employed in the same Opto-protein to create variants with different properties and functionalities (Li et al., 2020; Romano et al., 2021). In another work, Tichy et al. created a library of modular optogenetic domains for light-induced homodimerization, heterodimerization, oligomerization, and dissociation with different wavelengths to aid in the development of new light-sensitive proteins or signaling cascades (Tichy et al., 2019).

## 4 Truncation of the Photosensory and Interaction Domains, Removal of Cellular Signals

Along with the Opto-protein design strategy, the choice of the photosensory protein, and the availability of the corresponding chromophore, further analysis of the domain structure might be required for a functioning light regulator. Photoregulatory domains are often derived from natural multidomain proteins. When used for protein engineering purposes, it might be beneficial to identify and separate the domain of interest (dimerization/multimerization for spatial control, conformational change for allosteric control) from other domains that are present in the native photosensory protein. The truncation position of the photoregulatory domain can be crucial for the function of the newly designed optogenetic proteins and its selection requires careful evaluation of structural and functional units of the protein. In general, such truncations can have multiple purposes. First, the elimination of unnecessary domains might improve overall light-induction properties. Second, unwanted functions are removed (transport, interaction with other cellular or external signals, etc.), thus creating a more orthogonal light system and less interference with the fused functional domains. Third, a reduced size of the Opto-protein poses less burden to the cell for its expression and it results in smaller genetic constructs which could be important for specific applications (e.g. packaging into viral vectors). Fourth, altered protein structures may be preferable for certain protein fusions (e.g. N- and C-terminal proximity of the photoregulatory domains for split protein fusions or spatial positioning of the effector domains).

### 4.1 Shrinking and Adapting the Photosensory Domain

One of the first examples of photoreceptor truncation for a synthetic light regulator was shown by Ni et al. In this work, either full-length PhyB or the truncated PhyB(NT) version was fused to a Gal4 binding domain (Ni et al., 1999). The second part of the split two-hybrid system consisted of the phytochrome-interacting factor (PIF3) fused to the Gal4 activation domain. Light-induced binding was evaluated through an *in vitro* interaction assay. They found that photoactivated full-length PhyB is strongly bound by PIF3, but that also the N-terminal domain of PhyB is sufficient for moderate binding. PhyB(NT) with amino acid residues 1–621 was further used by Shimizu-Sato et al. for an early example of light-induced gene expression regulation in yeast, again using the same yeast two-hybrid assay that consists of the Gal4 binding domain fused to PhyB(NT) and the Gal4 activation domain fused to PIF3 (Shimizu-Sato et al., 2002). In a benchmarking study, also using a yeast two-hybrid system, Pathak et al. observed that PhyB(NT) leads to a much-increased output expression compared to full-length PhyB (Pathak et al., 2014). Truncations of the PhyB interacting proteins PIF3 (524 residues) and PIF6 (363 residues) to 100 amino acid residues (PIF3_APB_ and PIF6_APB_ respectively) were functional but led to decreased expression levels (Pathak et al., 2014). Thus, the possibility and potential advantages for the reduction of the PIF3 and PIF6 DNA-binding transcription factors to reduced interaction domains were successfully shown.

Similarly, truncations were also performed to change and improve the light-induced binding of *Arabidopsis thaliana* cryptochrome 2 (AtCRY2) to its binding partner cryptochrome-interacting basic-helix-loop-helix protein CIB1 which functions as a DNA-binding transcription factor. CIB1 interacts with CRY2 through blue light-stimulation to promote floral initiation in *Arabidopsis* (Liu et al., 2008). By considering only the light-responsive N-terminal photolyase homology region (PHR) that binds the chromophore, Kennedy et al. could increase light-induced gene expression using a yeast two-hybrid assay that consists of the Gal4 binding domain fused to Cry2 and a Gal4-CIB1 chimera (Kennedy et al., 2010). Dark state expression was also increased in the truncated CRY2PHR compared to full-length CRY2. A truncation of the interacting partner CIB1, called CIBN (from 335 to 170 amino acid residues), which is missing the conserved basic helix-loop-helix domain, also showed light-inducibility. In a subsequent study, CIB1 could be further truncated to an 81 amino acid residue fragment (CIB81) with similar light-induced expression properties as CIBN (Taslimi et al., 2016). In the same study, also two CRY2 truncations (residues 1–515 and 1–535) were tested that show an increased reporter expression level compared to full-length CRY2. Interestingly, CRY2 (535) showed lower self-association compared to the wild-type protein.

Size reduction and increased performance through photosensor truncations were also shown by Zhou et al. The design of the Opto-protein was based on a Gal4 two-hybrid system (see above) in combination with photoreceptor PhyA. Different transcriptional activators (VP16, VPR, P65-hsF1-VP64, p65-VP64, and VP64) and PhyA interacting proteins (FH1, FHL, and PIF3) were tested, out of which VP64 as a transcriptional activator, and FHY1-PhyA interaction protein showed the best switching performance. To enhance the light-induced expression, different previously described truncations of PhyA (Hiltbrunner et al., 2006) were tested. Out of these PhyA versions, only a truncated photoregulator containing the 617 N-terminal amino acids sufficiently activated trans-gene expression following cotransfection with the transactivator FHY1–VP16 (Zhou et al., 2021). In addition, the reduction in size from 1,126 amino acids of full-length PhyA to 617 amino acids in the truncated version enabled adeno-associated virus (AAV) packaging. This can be of particular relevance for the use of optogenetics in therapeutics, as AAV is a clinically approved vector for *in vivo* gene therapies in tissues and organs (Zhou et al., 2021).

Similarly, Kaberniuk et al. reduced the size of IsPadC BphP, a near-infrared-responsive protein, which enabled its AAV packaging (Kaberniuk et al., 2021). BphP binds the chromophore BV and contains an N-terminal photosensory core module, consisting of a PAS (Per-ARNT-Sim), GAF (cGMP phosphodiesterase/adenylate cyclase/FhlA transcriptional activator), and PHY (phytochrome-specific) domains, and a C-terminal effector domain (Chernov et al., 2017; Kaberniuk et al., 2021). Kaberniuk et al. created a chimera using the DBD of repressor LexA408 (Thliveris et al., 1991) and only the photosensory core module of IsPadC by excluding its cyclase effector domain.

The previously discussed examples thus truncated or eliminated sub-domains to alter the dimerization properties of the photosensitive domains. However, truncations were also employed to improve specific functions of photosensors or to change their outputs. For example, Nakajima et al. condensed the photosensory CcaS signaling kinase of the previously mentioned TCS CcaS/R by removing the two PAS domains of unknown function from the CcaS sensor kinase, which are located between the GAF and the HK domains of the protein (Nakajima et al., 2016). The remaining domains were connected by different truncated linker regions. While some of these “miniaturized CcaSs” that lack the PAS domains, showed similar activity compared to the wild-type sensor protein, also a version with higher expression compared to the wild-type sensor was found. In addition, two versions exhibited inversed light-inducibility compared to the native system. In these variants, red light activates gene expression which is otherwise inactivating in the native protein.

Truncations for an altered function of the photoreceptor were also used to enhance or reduce clustering of the photosensory domain of CRY2. As previously described, CRY2 undergoes homo-oligomerization upon blue light stimulation in addition to binding of its interaction partner CIB1. While CRY2-CIB1 interactions were used for reconstitution of split proteins, homo-oligomerization of CRY2 was exploited for cell regulation via clustering (both strategies are described in section 3.2). This dual-function could lead to issues of unintended homo-interaction induced within the same protein species of CRY2, in addition to the hetero-interaction of CRY2-CIB1. This motivated a study by Duan et al., which uncovered that charged residues at the N-terminus of CRY2 are critical for light-induced CRY2-CIB1 dimerization, while electrostatic charges at its C-terminus affect light-induced CRY2 homo-oligomerization. To better separate the two functions of CRY2, the CRY2 PHR domain (amino acids 1–498) was truncated to remove the positively charged N-terminus with lysins at amino acid positions 2, 5, and 6. This variant CRY2 (Δ2–6) showed a reduced affinity for CIB1, while oligomerization was similar to the wild-type Cry2 PHR (Duan et al., 2017). Also the C-terminus of CRY2 PHR contains three charged amino acid residues (arginines at positions 487 and 489; and glutamine at position 490) and their truncation in CRY2 (Δ487–498), CRY2 (Δ488–498), and CRY2 (Δ489–498) abolished cluster formation. However, CRY2 (Δ490–498), in which the glutamine at position 490 is deleted, exhibited aggregation, which led the authors to conclude that oligomerization is suppressed by glutamine 490, but enhanced by arginine 489. Although these variants showed no oligomerization, light-mediated CRY2–CIB1 dimerization was preserved (Duan et al., 2017). Thus, truncations at the N-terminus led to CRY2 variants that show oligomerization and reduced affinity for CIB1, while C-terminally truncated variants exhibited reduced clustering and similar affinity than the wild-type for CIB1.

Together with truncations that enable the adaptation of photosensor domain properties, mutations were described that alter certain properties of the photosensory domain. For example, in addition to the Cry2 truncations described in the previous paragraph, Duan et al. also identified that C-terminal negatively charged amino acids can further reduce clustering, while C-terminal positive charges can enhance it. Previously identified CRY2 (E490G) (Taslimi et al., 2014) as well as CRY2 (E490R), CRY2 (E490H) and CRY2 (E490K) showed increased protein aggregation (Duan et al., 2017). In contrast, clustering of the previously described truncation variant CRY2 (1–488) could be decreased further with additional negatively charged amino acids. Successive addition of negatively charged amino acid residues showed that this effect plateaued at 4 additional residues. These optimizations led the authors to arrive at an optimized CRY2 version named CRY2high and CRY2low with elevated or suppressed oligomerization respectively.

Multiple other property-modifying mutations have been described for photosensory domains, including ones that render the photosensory domain constitutively active or inactive, which is especially useful for testing new Opto-protein designs or can serve in experimental controls. On the example of CRY2, the FAD-deficient light-insensitive D387A mutant does not homo-oligomerize (Shao et al., 2020), and mutant CRY2 W374A exhibits constitutive homo-oligomerization activity *in vitro* and *in vivo* (Li et al., 2011). Such constitutive mutations have also been identified in other photoreceptors (e.g. Y276H in PhyB (Hu et al., 2009)). Also mutations that alter kinetic properties were described (e.g. (Taslimi et al., 2016) for mutations in CRY2).

In addition to modulating the oligomerization or binding of photosensory domains to their interaction partners in CRY2, mutations of photosensory domains were used to engineer multimerization states. For example, the heterodimerizing “Magnet” photosensory domains were developed based on the homodimerizing VIVID domain by engineering its homodimer interface, termed Ncap (Kawano et al., 2015). On the basis of their electrostatic interactions, one part of the heterodimerization system contains positively charged arginine amino acid residues at position 52 (positive Magnet; pMag; I52R and M55R) while the complementary protein domain contains a negatively charged aspartic acid at amino acid residue 52 site (negative Magnet; nMag; I52D and M55G). In comparison to the homodimerization system, the heterodimerization system allows for the assembly of different domains for example in split proteins (see section 3.2).

### 4.2 Removal of Encoded Signals

Native photosensitive domains might not just comprise photosensory and effector domains, but can also encode cellular signals, such as localization sequences, that might be embedded into the photosensory or interaction domain. The removal of such signals might improve the function of synthetic Opto-proteins. For example, Kennedy et al. mutated the predicted nuclear localization signals in Cry2 and CIBN, which led to their cytoplasmic expression (Kennedy et al., 2010). However, for full-length CIB1 this modification caused punctate perinuclear localization. Also domain fusions can generate such signals in the sequences of the fused domains. For example, Gil et al. observed light-induced nuclear export of a chimera consisting of the photoreceptor AsLOV2 with a nanobody. The fusion of the domains unintentionally created a nuclear export sequence (NES) within the linking region of some constructs (Gil et al., 2020). Nuclear export could be prohibited by the removal of the NES through a short (three residues) truncation of the LOV domain. Thus, such case-specific effects might have to be considered for the design and engineering of novel Opto-proteins.

### 4.3 Truncation of Effector Domains

Similar to the photosensory domain, also the truncation of effector domains might be advantageous or even required for a functioning Opto-protein. These modifications are often particular to the cellular function and thus the protein that shall become light-controlled. In some regulators, light-sensitive domains replace the function of other domains from the native effector protein. Previously described domain swapping strategies employ such truncations of the effector proteins and include a photosensitive domain instead of the native sensory domain. Similarly, also strategies for other functional domains have been employed. For example, light-controlled membrane localization was implemented by using truncated signaling proteins (Levskaya et al., 2009). This exploits that recruitment to the plasma membrane is an activation mechanism of signaling proteins. Light-activation of Rho-family G-protein signaling through light-induced translocation was achieved by Levskaya et al. using the catalytic DH-PH domain (Dbl-homology (DH) and pleckstrin-homolog (PH) domain) of the RacGEF Tiam, amongst other Rho-family proteins (Levskaya et al., 2009). Along with the DH-PH domains, Tiam1 contains an additional PH domain and a Discs-large homology (DHR) region at the N-terminus. DHR domains have been implicated as protein-protein interaction motifs and the N-terminal PH domain was shown to be essential for membrane localization and the formation of membrane ruffles (Michiels et al., 1997). In addition, the N-terminal PH domain was previously replaced by the c-Src membrane localization domain which caused membrane localization and membrane ruffling (Michiels et al., 1997). Levskaya et al. created a membrane-bound light-sensitive membrane recruitment protein, which consists of PhyB tagged with a fluorescent protein and is localized to the plasma membrane by the C-terminal CAAX motif of Kras (Choy et al., 1999). The catalytic DH-PH domains were further fused to PIF3, which enables red/far-red controlled membrane recruitment and release.

The necessity for such effector protein truncations is not only relevant in the context of the Opto-protein but can also be necessary for the greater context of the organism it is used in, such as shown by Pathak et al. This work aimed at adapting and improving a Gal4 two-hybrid system from yeast, the organism for which it was developed, to mammalian cells. This system employs a split Gal4 transcription factor fused to CRY2 and CIB1 to control transcription in a light-regulated manner in yeast (Kennedy et al., 2010) (for details see section 4.1). The same system did not result in light-controlled transcription in mammalian cells. Pathak et al. observed that CRY2-BD fusion constructs showed functional loss and undergo clustering and clearing in the nucleus in the presence of light. These observations inspired further modifications that included truncating the Gal4 DNA binding domain (1–147 amino acid residues) by removing residues 66–95, necessary for dimerization. In these constructs, light-induced oligomerization of CRY2 substitutes for the missing dimerization domain and restored activity to a large extent (Pathak et al., 2014). The optimized CRY2/CIB1 split Gal4 system with Gal4ΔDD (Gal4 residues 1–65) showed minimal light-induced localization changes, and greatly enhanced light-stimulated activity with low leakiness activity in the dark.

### 4.4 Fusion of Sensor and Effector Domains

Domains used for creating chimeric Opto-proteins can either be implemented containing the entirety or parts of the native linker of the individual domains (Ohlendorf et al., 2016; Romano et al., 2021). An example for the use of the native linker region of the effector protein being crucial for functionality was shown for the construction of the transcriptional photoregulator BLADE (blue light-inducible AraC dimers in *E. coli*) (Romano et al., 2021). BLADE consists of the DNA-binding and activation domain of AraC and the VVD LOV domain. Blue light-induced homodimerization of the protein enables binding of the corresponding araBAD promoter and activation of transcription. Different fusions were tested that contained the natural AraC linker region, truncations thereof, and different synthetic linkers in addition to the native one. Variants lacking the linker region showed no function, and the highest activity was seen with versions containing the full linker region with and without additional synthetic linker sequences. A different approach for linkage of domains relies on primer-aided truncations of the sensory and effector domains. The PATCHY strategy (primer-aided truncation for the creation of hybrid proteins) generates defined libraries of receptor variants that differ in length and composition of the linker regions. This strategy was applied to the previously described YF1 light-responsive histidine kinase. Here, residues 1–147 of BsYtvA, comprising the LOV domain and linker, and residues 255–505 of BjFixL, containing the linker region and the C-terminal DHp/CA effector, were fused through PATCHY to create variants with different properties (Ohlendorf et al., 2016). While these examples demonstrate the influence of truncations of natural protein linkers, they will be discussed in more detail also in the following section on effective linking of sensor and effector domains.

### 4.5 Tighter Coupling Between Sensor and Effector Domains

Usually, steric interference of different Opto-protein domains has to be avoided and/or domains precisely aligned for functionality. Certain design strategies of intramolecular optogenetic control require tight coupling of the involved protein domains in addition. One such example was shown by Gil et al. for the development of Opto-nanobodies (OptoNBs), which consist of chimeras of the light-sensing domain from *Avena sativa* Phototropin 1 (AsLOV2) incorporated into the single variable domain of camelid antibodies (Gil et al., 2020). This enables the binding of OptoNBs to proteins of interest which is either enhanced or inhibited upon blue light illumination. During the optimization process, sequences at N- and C-termini of AsLOV2 were truncated to create sLOV, with the intention to remove nuclear export signals (described in section 4.2), however, the authors also observed enhanced light-induced binding changes in 5 of 6 cases of the OptoNBs, presumably through tight coupling.

Another example of improved coupling of sensor and effector domains was shown with the development of optoWnt (Rosenbloom et al., 2020). This Opto-protein is composed of a fusion of the C-terminal domain of LRP6c to the CRY2 PHR. The main difference from the previously described β-catenin signaling system (see section 3.2) was the removal of a fluorescent protein, which, in the original work by Bugaj et al. was placed between LRP6c and CRY2 PHR to visualize cluster formation. This domain removal dramatically enhanced light-induced β-catenin activity, presumably due to improved orientation of the downstream effector domains.

While truncation, transferability, and exchangeability of photosensory domains have been demonstrated, such as in the discussed examples, it should also be mentioned that the individual domains of a protein naturally also interact with each other, which can have an impact on its function. For example, C-terminal truncations of PhyB alter its spectroscopic and kinetic properties, which can lead to a hypsochromic shifted (blue shifted) absorption maximum and faster thermal reversion from the light-activated to the inactive state (Burgie et al., 2017). In another study, truncations led to a change of the function of the Opto-protein such as an inversion of the output of the photosensory domain altogether, as already discussed for the miniaturized CcaSs (Nakajima et al., 2016). Not always can such changes be predicted and thus have to be thoroughly evaluated.

## 5 Interdomain Linkers and Their Influence on Opto-Protein Function

While it is clear that both the photosensory domain as well as the effector domain are essential components of newly created recombinant optogenetic protein fusions, it might be less obvious that also the amino acid residues linking the individual domains can be essential for a functioning Opto-protein. This includes effects such as impaired protein folding, which can be a result of direct fusion without a linker, or might cause low expression of the chimera, or impaired bioactivity (Chen et al., 2013). Linkers and the steric positioning of the sensor and effector domains do not just determine if and how well optogenetic fusion proteins work, but can play an important role in defining specific properties of the hybrid protein.

In general and according to their structures, amino acid residue linkers can be classified into either flexible linkers, that provide movement between the domains and typically consist of small non-polar (glycine) or polar (serine, threonine) residues, rigid linkers, in which stiff α-helical structures may act as rigid spacers between domains, or *in vivo* cleavable linkers, which are usually less relevant for Opto-protein engineering (Chen et al., 2013). Linker lengths occurring between natural multidomain structures have an average length between 4.5 ± 0.7 for small and 21 ± 7.6 residues for long linkers, with increasing solvent accessibility and decreasing hydrophobicity for increasing linker length (George and Heringa 2002; Chen et al., 2013). In naturally occurring linkers, generally polar uncharged or charged residues are preferred, in particular amino acid residues threonine, serine, proline, glycine, aspartic acid, lysine, glutamine, asparagine, alanine, arginine, phenylalanine, and glutamic acid (Argos 1990; George and Heringa 2002; Chen et al., 2013). While proline residues are thought to increase the stiffness and structural independence of the linkers, small, polar amino acids, such as threonine, serine and glycine might provide good flexibility due to their small sizes, and maintain stability through the formation of hydrogen bonds with water (Chen et al., 2013). Further, α-helix or coil secondary structures are most common in natural linkers (Argos 1990; George and Heringa 2002). α-helix linkers (e.g. in the form of A (EAAAK)_n_A with *n* = 2-5 or (XP)n, with X preferably Ala, Lys, or Glu) might aid with correct folding, as their structure is formed rapidly, which could reduce non-native interactions with other domains, and their rigid structure might be used as a spacer to separate domains and reduce unfavorable interactions as well as to generate spatially defined structures (Chen et al., 2013).

The importance of hybrid protein linker regions was nicely demonstrated by Ohlendorf et al., 2016. The authors applied systematic modification and analysis of the linker between the photosensory domain of *Bs*YtvA and the *Bj*FixL effector domain of YF1, which comprises the sensory part of their blue-light sensing two-component system for transcription regulation (Ohlendorf et al., 2012). Using primers to truncate the linking region between the two domains revealed that variants with 7n or 7n+5 linker residues were repressed with blue-light, whereas variants with 7n+1 residues were blue-light-activated, as well as constitutive variants (Ohlendorf et al., 2016). This periodicity might be attributed to a continuous α-helical coiled−coil conformation and the resulting angular orientation that changes by 103° per residue. Also, functional variants were identified that span a linker length of 4–50 amino acid residues (distances of separation between ∼6 and 75 Å), which might also be accounted to the rigid linker structure.

While linker lengths may be less important than structural considerations such as angular positioning in rigid α-helical coiled-coils (Ohlendorf et al., 2016), linker length in flexible linkers was shown to be an important determinant of photoreceptor activity. This was observed by Romano, Baumschlager et al., where two different photosensitive LOV domains (VVD and VfAu1) were individually fused to the DNA-binding and transcriptional activation domain (DBD) of the transcription factor AraC (Romano et al., 2021). Thus, the native arabinose-binding and dimerization domain (DD) of the AraC protein is replaced with a photosensitive domain. While the direct fusion of VVD and AraC(DBD) showed no light-inducibility, constructs that contain the native linker connecting the AraC(DD) and the AraC(DBD) were functional. Extending this linker with up to 7 additional amino acid residues, consisting of glycine and serine or alanine, for the VVD fusions showed similar light-inducibility than just the natural linker alone. However, longer additional linkers led to reduced light-induced activation. Similar results were observed when VfAu1 was used as photoregulatory domain.

The specific amino acid residues occurring at the separation site of split proteins can also be considered. For example, for construction of the Opto-T7RNAPs (Baumschlager et al., 2017; Dionisi et al., 2021) split sites within the T7 RNA polymerase in surface-exposed loops between amino acid residues commonly found in natural linkers, were chosen (Baumschlager et al., 2017). Short synthetic linkers were then added for fusion of the polymerase split fragments to the light-inducible “Magnet” heterodimerization system.

## 6 Finding the Sweet Spot of Opto-Protein Concentration

**FIGURE 3:**
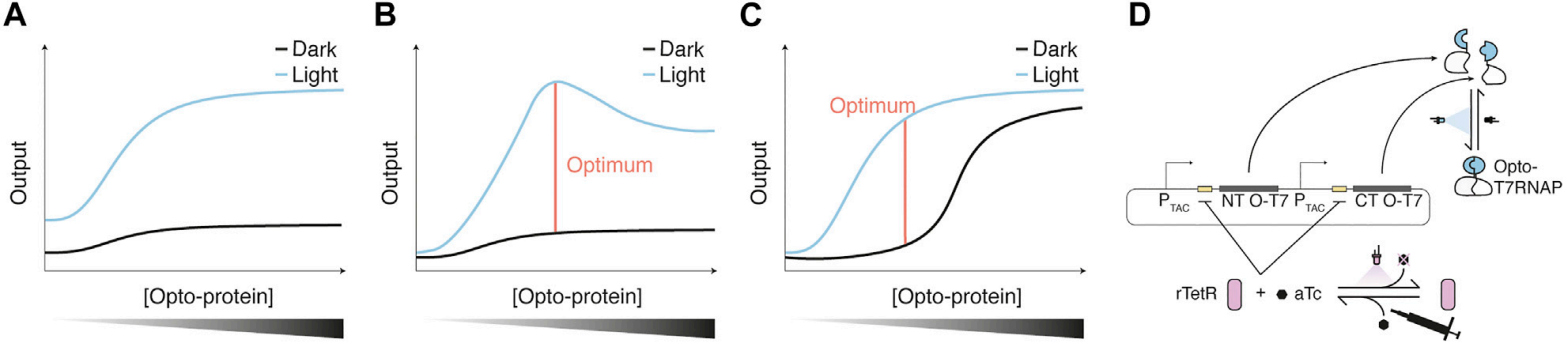
Optimization of Opto-protein concentration **(A–C)** Potential scenarios encountered for light-induced and dark-state output/activity obtained with increasing Opto-protein concentrations. Optimal regulator expression is defined as the fold change of dark to light-induced reporter expression. In scenario one **(A)**, dark and light-induced output increase proportionally, therefore the fold change is maintained for various regulator concentrations. Scenarios two **(B)** and three **(C)** show two examples in which the fold change is maximal at specific Opto-protein concentrations. Scenario two could be caused by the burden of the regulator expression which could lead to a reduction of the output, while in scenario three, a high regulator concentration might lead to increased dark state activation due to, for example, molecular crowding. **(D)** The concentration of the Opto-protein can also be adjusted throughout an experiment/biological process via a dual control scheme as shown for the Opto-T7RNAP, whose expression was controlled through the repressor rTetR (Baumschlager et al., 2020). rTetR binds tetO operator sequences of a corresponding promoter with anhydrotetracycline (aTc) bound, which represses transcription of the Opto-protein regulator. aTc can be inactivated with UVA light, leading to an induction of Opto-T7RNAP expression, which in turn can be activated with blue light. This regulation scheme was used to reduce the dark state activity of Opto-T7RNAP in the presence of aTc.

Not only the design of the Opto-protein itself but also its concentration in the cell can be critical for optimal functionality. The focus of such an optimization might depend on the requirements of the application the Opto-protein shall fulfill. For example, studying the function of a gene of interest may require a dark state activity that is not detectible or does not lead to a certain phenotype, while light activation still has to induce a phenotypic change. In other cases, the difference between on and off states, so a high dark-to-light fold change, might be crucial. In other cases that simply require strong activation, optimization could favor a high light-induced activity potentially at the cost of higher dark state activity. In some cases, the output can simply be adjusted through the concentration of the Opto-protein itself (Figure 3). An example for a similar increase of dark and light-induced activity with increased Opto-protein concentration was shown for Opto-T7RNAPs (Figure 4 left panel of (Baumschlager et al., 2017)). In addition, considerations such as cellular burden or toxic effects imposed by the Opto-protein or its output might also influence the expression strategy of the Opto-protein. Depending on the nature of the Opto-protein and its design, intermediate concentrations might lead to favorable light/dark ratios. (Figures 3B,C).

An example for optimization of Opto-protein performance via tuning of its concentration was shown by Schmidl et al. for the previously described CcaS/R light-inducible TCS (Tabor et al., 2011). For this, both the sensor kinase as well as the response regulator were expressed using different RBS strengths. Especially the expression level of CcaR led to an increase in the fluorescent output created by the TCS under both red and green light. The authors found that intermediate expression levels, especially of the response regulator, led to the highest fold-changes of light-induction. This optimization thus resulted in lower leakiness and a higher fold-change of the TCS (Schmidl et al., 2014).

Similarly, different ribosome binding site (RBS) strengths were used for tuning a single component expression system (Li et al., 2020). Here, a transcription regulator called eLightOn was developed, which consists of the LexA408 DNA binding domain (see section 4.1) and the RsLOV light-inducible homodimerization domain. With increasing expression strength of the Opto-protein, both the light-induced as well as the dark expression levels increased, up to a point where dark and light-induced expression levels were similar. Thus, intermediate expression levels of the Opto-protein led to the highest fold-changes.

Such an expression level tuning was also performed for BLADE (see section 5). For this, the Opto-protein was expressed from a chemically-inducible promoter to examine the dark and light-activated function of BLADE at different concentrations. The expression level generated by the chemical inducer was mapped to a set of constitutive promoters, which enables fixing the expression to desired levels of dark and light-induced expression. Also for BLADE, both dark and light-induced expression increased with the concentration of the Opto-protein, with the highest fold-changes at intermediate Opto-protein concentrations. (Figures 3B,C). Further, also the overall maximal light-induced expression generated by certain BLADE constructs was highest at intermediate Opto-protein concentrations (Romano et al., 2021).

A different approach aims at additional chemical regulation of the Opto-protein activity/concentration during the process to improve properties such as fold-change, overall expression level, and dark state expression. This was shown in a combination of a light-sensitive chemical with an Opto-protein. For this, the expression of the Opto-T7RNAPs (see section 5) was controlled through the light-sensitive chemical anhydrotetracycline (aTc), thus creating a dual-control system (Baumschlager et al., 2020). The expression of Opto-T7RNAP is regulated by rTetR, which binds tetO operator sequences in the presence of aTc. UVA light inactivates aTc, which leads to unbinding of the rTetR repressor from the promoter and expression of the Opto-T7RNAP. (Figure 3D). This concept was used to get a lower dark state expression of Opto-T7RNAP in the presence of aTc, and a high light-induced expression when aTc is not present or was inactivated (Baumschlager et al., 2020).

## 7 Summary

Engineering a specific cell function light regulatable requires several considerations. As in most biological engineering approaches, one important point is context, such as the target cell, which determines the availability of chromophores utilized by photosensitive domains or the possibility for the transfer of photoregulatory circuits from other organisms. For the direct transformation of a protein of interest into a light-inducible one, it needs to be evaluated which form of regulation (intermolecular or intramolecular) might be most suitable. Careful structural and functional analysis of the photosensory domain components, as well as their linkage to the effector domains and the concentration of the resulting Opto-proteins, can play critical roles for optimization steps. As in all biology engineering approaches, the effects of the induction, which might be less obvious for light as an inducing agent, and the expression of the chimeric proteins, need to be carefully evaluated for crosstalk and other unwanted effects. Ultimately, the ability for screening the function of interest will set boundaries for the testing of different Opto-protein designs and optimization steps. Considering the points raised in this review might aid in the development of new Opto-proteins as well as the optimization of existing ones for creating a brighter future.
